# Stent edge vascular response and in-stent geometry after aerobic exercise

**DOI:** 10.1007/s12928-020-00655-5

**Published:** 2020-03-09

**Authors:** Maria Dalen Taraldsen, Vibeke Videm, Knut Hegbom, Rune Wiseth, Erik Madssen

**Affiliations:** 1 Department of Circulation and Medical Imaging, NTNU, Norwegian University of Science and Technology, Trondheim, Norway; 2 Department of Clinical and Molecular Medicine, NTNU, Norwegian University of Science and Technology, Trondheim, Norway; 3 Department of Immunology and Transfusion Medicine, St. Olavs University Hospital, Trondheim, Norway; 4 Clinic of Cardiology, St. Olavs University Hospital, Trondheim, Norway

**Keywords:** Aerobic exercise, Stent edge, Drug-eluting stent

## Abstract

The purpose of the present study was to investigate the edge vascular response in patients treated with second-generation drug-eluting stents (DES) after 3 months of aerobic exercise intervention. Thirty-two patients with significant coronary artery disease underwent percutaneous coronary intervention with DES implantation prior to randomization to aerobic interval training (AIT, 14 patients) versus moderate continuous training (MCT, 18 patients). Plaque changes were assessed using grayscale and radiofrequency intravascular ultrasound at baseline and follow-up. The main endpoints were changes in plaque burden and necrotic core content in the 5-mm proximal and distal stent edges. Plaque burden in the distal stent edges decreased significantly in both groups (AIT: − 3.3%; MCT: − 0.4%, *p* = 0.01 for both), and more in the AIT group (*p* = 0.048). Necrotic core content decreased significantly in the distal stent edges in both groups (− 2.1 mm^3^ in AIT, − 0.3 mm^3^ in MCT, *p* = 0.01 for both), and more in the AIT group (*p* = 0.03). There were no significant changes in proximal stent edges or in in-stent geometry at follow-up. In this small study of patients treated with DES implantation, 3 months of aerobic exercise training demonstrated decreased plaque burden and necrotic core content in the distal stent edges, with larger reductions in the AIT group.

## Introduction

Percutaneous coronary intervention (PCI) with implantation of second-generation drug-eluting stents (DES) in patients with coronary artery disease (CAD) reduces in-stent restenosis and need for repeat revascularization compared to bare-metal stents [1, 2]. Despite the benefits of DES, adverse long-term effects after stenting may occur, such as in-segment restenosis requiring repeat revascularization. The edge vascular response (EVR) in the 5 mm coronary artery segment proximal and distal to the stent edges, includes tissue proliferation, arterial remodeling and late luminal loss that may contribute to restenosis [3].

Physical activity is pivotal in the secondary prevention of CAD [4]. A vast amount of evidence has established that high cardiorespiratory fitness measured as peak oxygen uptake (VO_2peak_) is associated with a reduced risk for CAD and all-cause mortality [5]. Previous studies have shown that aerobic interval training (AIT) is superior to moderate continuous exercise (MCT) with respect to increasing VO_2peak_ in patients with CAD. Furthermore, high-intensity exercise is also associated with larger effects on several surrogate markers for atherosclerosis, such as endothelial function and inflammation [6–8].

However, limited data are available on both short- and long-term effects of aerobic exercise on the EVR and in-stent vascular responses after DES implantation. Some previous angiographic studies suggest beneficial in-stent effects from exercise with reduced late luminal loss [9–11]. However, we are not aware of previous studies assessing exercise-induced effects on in-stent geometry or the EVR using intravascular ultrasound (IVUS).

The aim of the present study was, therefore, to investigate the EVR and in-stent geometry using grayscale and radiofrequency IVUS in patients with CAD undergoing PCI with implantation of second-generation DES and randomized to two different aerobic exercise interventions, i.e. aerobic interval training (AIT) versus moderate continuous training (MCT).

The primary endpoints were defined as changes from baseline to follow-up in plaque burden and necrotic core content in the proximal and distal stent edges. We hypothesized wall shear stress (WSS) during exercise to increase more in the AIT group compared to the MCT group due to differences in exercise intensity, and therefore, hypothesized more beneficial EVR changes in the AIT group compared to the MCT group.

## Methods

This study was a post hoc analysis of data from a completed randomized controlled trial (clinicaltrials.gov identifier NCT01228201) from the Clinic of Cardiology, St. Olav`s University Hospital and NTNU-the Norwegian University of Science and Technology, Trondheim, Norway. The study investigated the effects of two different aerobic exercise interventions, i.e. aerobic interval training (AIT) versus moderate continuous training (MCT), in non-stented coronary segments [12]. This was a single-center, open, parallel, randomized controlled trial performed between December 2010 and April 2012.

The study protocol has been described in detail elsewhere [12]. This was a pilot study and no historical data were, therefore, available for sample size calculation. Thus, we had to anticipate a priori a difference in cross-sectional plaque area between the exercise groups of 40%, which corresponds to 2.7 mm. To show this difference with a power of 80% using a 2-sided t-test with alfa = 0.05, 19 patients in each group, i.e. 38 patients in total, were needed. Forty-one patients were randomized in the first trial, and finally 36 patients with angina pectoris or non-ST elevation acute coronary syndrome requiring revascularization with stent implantation were included. A flow diagram of enrollment, randomization, follow-up and data analyses in the randomized controlled trial has been previously published [12].

All patients received optimal medical treatment and standard in-hospital care. In the present study, four patients receiving bare-metal stents were excluded to minimize the stent variability; one AIT patient and three MCT patients, leaving a total of 32 patients (14 AIT patients and 18 MCT patients, respectively) for analysis. Of the 32 patients, one AIT patient and three MCT patients had two target lesions in the index artery. We randomly selected one of the two stents from the four patients with two stents to avoid a design with within-patient correlations between stents.

Patients in the study used either 40 mg of simvastatin or 40 mg of atorvastatin at inclusion. There were no differences between the exercise groups with respect to the use of these two different statins, and during the study the statin regimen remained unchanged for all patients.

Three types of second-generation DES were used: the Xience Everolimus-Eluting Stent, the Resolute Integrity Zotarolimus-Euting Stent and the Promus Premiere Everolimus-Eluting Stent (Table 1). The Eagle Eye Platinum IVUS 20 MHz probe (Volcano Corporation, Rancho Cordova, CA) was used for obtaining grayscale intravascular ultrasound (GS-IVUS) and radiofrequency intravascular ultrasound (RF-IVUS) data in the index artery. A pullback rate of 0.5 mm/s was used (Volcano R100 pullback device) collecting the RF backscatter data at every R peak in the electrocardiogram. There were no complications related to imaging during the study.

**Table 1:** Baseline characteristics of the study population

	AIT (*n* = 14)	MCT (*n* = 18)	*P* value
Age, years	56 (51–61)	61 (56–65)	0.60
Male/female	13/1	12/6	0.08
Current smokers, *n* (%)	3 (21%)	3 (17%)	0.03
Hypertension, *n* (%)	8 (57%)	9 (50%)	0.69
Diabetes, *n* (%)	5 (36%)	2 (11%)	0.10
HbA1c (%)	5.8 (5.5–6.5)	5.7 (5.5–6.1)	0.28
Hypercholesterolemia, *n* (%)	4 (29%)	7 (39%)	0.54
Triglycerides, mmol/L	1.1 (0.9–1.4)	1.2 (0.9–1.5)	0.90
High density lipoprotein cholesterol, mmol/L	1.1 (1.0–1.4)	1.3 (1.1–1.4)	0.31
Low density lipoprotein cholesterol, mmol/L	2.3 (2.1–2.6)	2.3 (2.0–2.8)	0.56
Total cholesterol, mmol/L	4 (3.8–4.8)	4.4 (3.9–4.8)	0.34
Prior myocardial infarction, *n* (%)	1 (7%)	4 (22%)	0.24
Prior PCI^a^, *n* (%)	3 (21%)	5 (28%)	0.68
Current diagnosis, *n* (%)			
Angina pectoris	8 (57%)	10 (56%)	0.98
Non-ST elevation acute coronary syndrome (NSTE-ACS)	6 (43%)	8 (44%)	
Severity of coronary artery disease, *n* (%)			
1-vessel disease	10 (71.5%)	10 (56%)	0.25
2-vessel disease	3 (21.5%)	8 (44%)	
3-vessel disease	1 (7%)	0 (0%)	
Target vessel location, *n* (%)			
Left anterior descending artery	10 (71.5%)	9 (50%)	0.38
Circumflex artery	1 (7%)	4 (22%)	
Right coronary artery	3 (21.5%)	5 (28%)	
Type of drug-eluting stent (DES) implanted			
Xience	10 (66.7%)	16 (76%)	0.46
Resolute	4 (26.7%)	5 (24%)	
Promus	1 (6.6%)	0 (0%)	
Lesion length, mm	18.4 (13.0–21.5)	12.0 (10.0–16.5)	0.03
Stent length, mm	27.8 (20.7–37.6)	19.2 (15.7–24.5)	0.047
Stenosis diameter prior to PCI (%)	64.3 (55.0–75.0)	71.0 (62.5–79.5)	0.33
Minimal luminal diameter prior to PCI, mm	0.97 (0.65–1.16)	0.99 (0.79–1.19)	0.81
Reference vessel diameter prior to PCI, mm	2.79 (2.48–3.13)	3.11 (2.82–3.35)	0.13
Medication, *n* (%)			
Acetylsalicylic acid (ASA)	14 (100%)	18 (100%)	NA^b^
Clopidogrel	14 (100%)	18 (100%)	NA
Statins	14 (100%)	18 (100%)	NA
Betablockers	11 (79%)	14 (78%)	0.95
Angiotensin-converting-enzyme inhibitor (ACEi) or Angiotensin II receptor blockers (ARB)	8 (57%)	10 (56%)	0.93

Continuous variables are given as medians with 95% confidence intervals in parenthesis

^a^Percutaneous intervention

^b^*NA* not applicable

Data were analyzed at an independent core lab (Krakow Cardiovascular Research Institute, Krakow, Poland) according to current recommendations for analysis and reporting of intravascular ultrasound and radiofrequency data) [13, 14]. All analysts were blinded to clinical data and randomization. Intravascular data were analyzed using QIvus software 2.1 (Medis Medical Imaging System, Leiden, The Netherlands). Matched coronary segments at baseline and follow-up were controlled manually after automatic detection using landmarks such as side branches, bifurcations and the implanted stent. Three regions of interest were defined: the stented coronary segment, the 5 mm artery segment proximal to the stent edge, and the 5 mm artery segment distal to the stent edge (defined as proximal and distal edges, respectively).

The following GS-IVUS parameters were analyzed: minimal lumen area, stenosis at minimum lumen area (percentage stenosis at the smallest cross-sectional lumen area detected in the analyzed segment), total atheroma volume (cross-sectional area of external elastic membrane minus cross-sectional area of lumen in the 5 mm segment) and plaque burden (the percentage atheroma volume in the whole 5 mm segment defined as plaque plus media area divided by the vessel area multiplied by 100). The RF-IVUS parameter fibrous tissue, fibro-fatty tissue, necrotic core and dense calcium were computed from the backscattered RF data and reported in absolute volumes and percentage volumes over the 5 mm long proximal and distal edge segment [15]. The quantitative coronary angiography parameter late lumen loss was defined as the angiographic minimal lumen diameter after PCI minus the minimal lumen diameter at follow-up.

Peak oxygen uptake (VO_2peak_) was calculated as the mean of the three highest VO_2_ measurements from a treadmill cardiopulmonary exercise test (Oxycon Pro Jaeger, Hoechberg, Germany). The peak heart rate (HR) was measured as the highest HR during the test.

Both the AIT and MCT groups met for supervised exercise training three times a week for 12 weeks. The length of the exercise protocol corresponds to the length of the standard cardiac rehabilitation program offered to patients in our health region. Patients were required to complete > 90% of planned sessions. Patients in the MCT group walked or ran lightly for 46 min at 70% of maximum HR. The AIT patients completed 10 min of warm-up followed by four times 4-min intervals at 85–95% of maximum HR, with 3 min of active rest at 70% of maximum HR between each interval and before terminating the session. The MCT and AIT exercise protocols were based on previous study protocols from the Cardiac Exercise Research Group at NTNU [6, 7, 16, 17].

### Statistical analysis

Data are expressed as medians with 95% confidence intervals in parentheses. Medians were preferred due to a small patient population and few normally distributed variables. SPSS (version 23.0, IBM) and Minitab (version 17.0) were used to analyze the data. Baseline characteristics were compared using the Mann–Whitney *U* test or the Chi-square test. Two-way ANOVA (analysis of variance) for repeated measures was used to analyze baseline and follow-up data, with focus on the p-values for overall parameter change by time (within-subject change) and time-by-group interaction (corresponding to difference in parameter change by time between the exercise groups). Model fit was evaluated using residual plots. If necessary, variables were logarithmically transformed to achieve appropriate fit. Because some variables were significantly different between the AIT and MCT groups at baseline, we also performed adjusted sensitivity analyses for the IVUS variables that showed significant changes by time. Thus, an additional ANOVA analysis was performed including either lesion length, stent length, or smoking as an adjustment. We also performed an adjusted sensitivity ANOVA analysis where all stents were included, i.e. without random selection of one stent from the patients with two stents. Because plaque characteristics might be influenced by lipid changes, we also performed sensitivity analyses including either statin type or changes in lipids from baseline to follow-up (low-density lipoprotein (LDL) cholesterol, high-density lipoprotein (HDL) cholesterol, triglycerides, or total cholesterol) as an adjustment. We also performed sensitivity analyses including HbA1c, diabetes or hypertension. Finally, we performed an adjusted sensitivity analysis for clinical presentation of CAD, i.e. stable angina vs. acute coronary syndrome.

## Results

Baseline characteristics of the study population are presented in Table 1. Patients in the AIT group had longer lesion lengths and longer stent lengths than the MCT group; otherwise, there were no significant baseline differences between the groups. The attainment level to the exercise protocols was excellent. All patients completed > 90% of planned sessions as required. There were no significant differences in LDL cholesterol, HDL cholesterol, triglycerides, total cholesterol or HbA1c at follow-up.

GS- and RF-IVUS data at baseline and follow-up in-stent edge segments are presented in Table 2. There was a significant decrease in plaque burden in the distal stent edge in both groups (*p* = 0.01), and more so in the AIT group (*p* = 0.048). The changes in plaque burden were − 3.3% (− 7.1,− 0.4) and − 0.4% (− 3.3, 2.6) in the AIT and MCT group, respectively.

**Table 2:** Grayscale and radiofrequency intravascular ultrasound data at baseline and follow-up in-stent edge segments

	AIT		MCT		*P* value^a^
	Baseline	Follow-up	Baseline	Follow-up	
Proximal edge segments					
Minimal lumen area (mm^2^)	6.8 (5.0–7.9)	6.8 (5.2–8.5)	7.0 (5.7–7.8)	7.5 (5.7–8.5)	0.08^†^ 0.06^††^
Total atheroma volume (mm^3^)	8.2 (4.2–12.6)	7.8 (4.0–12.3)	9.1 (6.2–10.2)	8.7 (6.7–10.5)	0.36^†^ 0.39^††^
Total vessel volume (mm^3^)	84.1 (58.0–104.4)	93.0 (59.8–108.3)	83.5 (64.8–98.6)	92.7 (71.4–98.2)	0.24^†^ 0.93^††^
Total lumen volume (mm^3^)	40.0 (32.7–46.0)	42.7 (34.7–53.4)	41.8 (34.4–50.3)	43.3 (29.4–53.5)	0.04^†^ 0.94^††^
Stenosis at minimal lumen area (%)	53.9 (41.3–70.8)	50.2 (38.1–68.3)	61.3 (58.8–62.6)	61.1 (56.3–64.2)	0.19^†^ 0.08^††^
Plaque burden (percent atheroma volume, %)	46.6 (37.6–61.9)	46.6 (31.0–60.2)	55.7 (46.6–57.1)	51.3 (43.4–55.8)	0.09^†^ 0.82^††^
Fibrous tissue (mm^3^)	11.6 (7.1–20.7)	12.7 (5.8–22.3)	12.6 (7.8–19.9)	12.3 (10.4–19.7)	0.98^†^ 0.31^††^
Fibro-fatty tissue (mm^3^)	2.0 (0.3–6.6)	2.1 (0.8–6.3)	2.3 (1.1–2.8)	2.4 (1.7–3.6)	0.53^†^ 0.81^††^
Necrotic core (mm^3^)	6.8 (1.6–9.5)	4.5 (1.5–8.8)	5.0 (3.1–10.8)	5.9 (2.8–11.0)	0.38^†^ 0.31^††^
Dense calcium (mm^3^)	1.7 (0.3–5.0)	1.6 (0.1–6.5)	1.2 (0.6–3.5)	1.7 (0.8–3.1)	0.91^†^ 0.95^††^
Fibrous tissue (%)	54.1 (49.0–70.5)	56.7 (40.9–76.6)	60.1 (52.8–65.4)	60.6 (52.9–64.4)	0.58^†^ 0.63^††^
Fibro-fatty tissue (%)	14.8 (5.1–18.0)	11.8 (7.0–16.4)	8.9 (6.4–12.9)	10.1 (7.9–15.0)	0.59^†^ 0.41^††^
Necrotic core (%)	21.2 (10.8–35.9)	20.4 (15.1–23.6)	22.8 (17.7–30.1)	21.2 (16.3–29.3)	0.20^†^ 0.52^††^
Dense calcium (%)	8.1 (2.1–14.3)	6.1 (3.3–19.7)	6.8 (2.5–13.5)	5.5 (4.3–12.8)	0.53^†^ 0.27^††^
Distal edge segments					
Minimal lumen area (mm^2^)	6.5 (4.9–6.8)	6.3 (5.2–7.0)	5.9 (4.7–6.9)	5.9 (5.6–7.1)	0.57^†^ 0.95^††^
Total atheroma volume (mm^3^)	3.8 (2.6–6.6)	3.7 (2.1–6.3)	5.3 (3.8–7.6)	5.3 (3.9–7.3)	0.15^†^ 0.22^††^
Total vessel volume (mm^3^)	56.2 (41.3–70.2)	53.3 (39.5–72.7)	58.3 (44.2–81.5)	61.6 (52.1–83.0)	0.40^†^ 0.09^††^
Total lumen volume (mm^3^)	35.7 (25.6–39.7)	36.2 (26.5–44.6)	37.6 (25.8–42.4)	38.7 (28.6–41.1)	0.03^†^ 0.56^††^
Stenosis at minimal lumen area (%)	40.0 (31.9–53.5)	37.2 (24.0–50.9)	51.5 (37.8–61.0)	49.2 (38.3–57.4)	0.03^†^ 0.32^††^
Plaque burden (percent atheroma volume, %)	39.6 (28.5–50.2)	36.7 (24.3–44.5)	42.8 (34.4–51.8)	42.1 (36.9–48.1)	0.01^†^ 0.048^††^
Fibrous tissue (mm^3^)	5.0 (0.7–10.9)	3.0 (0.8–11.6)	9.2 (4.5–13.6)	9.5 (5.9–18.0)	0.43^†^ 0.02^††^
Fibro-fatty tissue (mm^3^)	0.8 (0.04–1.3)	0.8 (0.1–2.1)	1.0 (0.5–1.4)	1.1 (0.5–2.7)	0.60^†^ 0.96^††^
Necrotic core (mm^3^)	2.7 (0.3–7.2)	1.4 (0.1–5.2)	2.9 (0.8–7.0)	2.3 (0.9–5.1)	0.01^†^ 0.03^††^
Dense calcium (mm^3^)	1.2 (0.1–2.9)	1.2 (0.02–1.5)	0.6 (0.1–1.2)	0.4 (0.1–1.0)	0.006^†^ 0.15^††^
Fibrous tissue (%)	60.7 (47.7–81.2)	70.1 (59.6–81.5)	63.4 (57.1–71.4)	67.9 (66.3–74.3)	0.02^†^ 0.53^††^
Fibro-fatty tissue (%)	6.2 (1.7–9.7)	7.1 (5.3–14.1)	8.7 (5.4–9.8)	10.0 (6.2–11.9)	0.04^†^ 0.53^††^
Necrotic core (%)	23.7 (8.0–32.1)	16.7 (3.5–24.8)	19.6 (16.1–29.3)	17.3 (13.1–21.7)	0.004^†^ 0.48^††^
Dense calcium (%)	6.7 (5.1–18.9)	3.2 (0.3–12.8)	3.5 (2.5–7.2)	2.4 (1.3–4.1)	0.048^†^ 0.43^††^

Data are given as medians with 95% confidence intervals in parenthesis

^†^Changes by time in both groups from baseline to follow-up

^††^Differences between the groups from baseline to follow-up (interaction effect)

^a^ANOVA (analysis of variance) for repeated measures was used to analyze baseline and follow-up data

Total lumen volume increased significantly both in the proximal stent edge (*p* = 0.04) and in the distal stent edge (*p* = 0.03) in both exercise groups, with no significant changes between the groups. There were no significant changes in total vessel volume in the proximal or distal stent edge within or between the two groups.

There was also a significant decrease in percentage stenosis at minimal lumen area, in the distal stent edge in both groups (*p* = 0.03), without any intergroup difference (*p* = 0.32). The changes in percentage stenosis at minimal lumen area were − 1.6% (− 9.0, 1.6) and − 2.5% (− 3.4, 1.7) in the AIT and the MCT group.

**Fig. 1:**
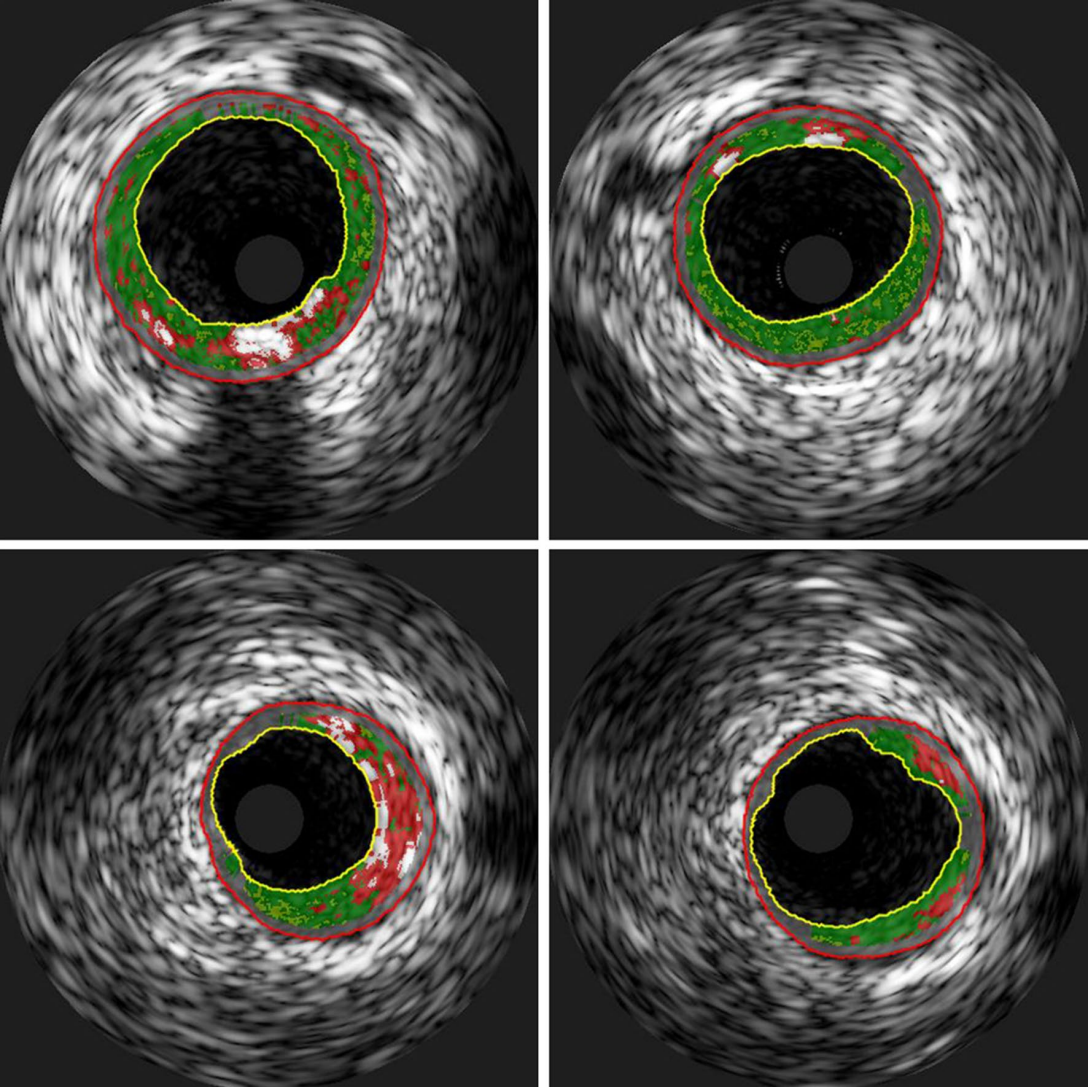
Radiofrequency IVUS cross-sectional images from 2 patients (both aerobic interval training) illustrating plaque changes from baseline (left) to follow-up (right). Both patients had a reduction in plaque burden and necrotic core volume. Green = fibrous tissue; green-yellowish = fibro-fatty tissue; red = necrotic core; and white = dense calcium

Necrotic core in the distal stent edges decreased in both groups (*p* = 0.01), and the percentage reduction was largest in the AIT group (*p* = 0.03). The changes in necrotic core volume were − 2.1 mm^3^ (− 3.2, 0.1) in the AIT group and − 0.3 mm^3^ (− 2.5, 0.1) in the MCT group. Figure 1 illustrates significant plaque changes in two patients undergoing AIT.

Dense calcium decreased significantly in both groups (*p* = 0.006) in the distal stent edge, without any intergroup difference (*p* = 0.15). The changes were − 0.8 mm^3^ (− 2.0, 0.4) and − 0.1 mm^3^ (− 0.6, 0.05) in the AIT and MCT group, respectively. There were no significant changes in any parameters in proximal edge segments at follow-up.

Fibrous tissue and fibro-fatty tissue in percentage volumes increased significantly in both groups (fibrous tissue: *p* = 0.02, fibro-fatty tissue: *p* = 0.04) in the distal stent edge, without any intergroup difference. Necrotic core and dense calcium in percentage volumes decreases significantly in both groups (necrotic core: *p* = 0.004, dense calcium: *p* = 0.048) in the distal stent edge, without any intergroup difference.

Inclusion of adjustment variables in the sensitivity analyses performed as described above did not alter the results, and none of the adjustment variables were significant. The adjusted sensitivity ANOVA analysis with all stents included gave the same results.

Table 3 presents quantitative coronary angiography and GS-IVUS data in the stented segment. There was a significant increase in-stent segment plaque volume within both exercise groups (*p* = 0.02), with no changes between the two groups. The stent segment plaque volume increased with 22.6 mm^3^ (− 6.7, 43.0) in the AIT group, and with 13.5 mm^3^ (− 8.2, 28.4) in the MCT group. There were no significant changes in-stent segment lumen or vessel volume, diameter stenosis, late luminal loss, or in neointimal volume. Changes in cardiorespiratory variables are shown in Table 4.

**Table 3:** Quantitative coronary angiography and grayscale intravascular ultrasound data at baseline and follow-up in-stent segments

	AIT		MCT		*P* value^a^
	Baseline	Follow-up	Baseline	Follow-up	
Diameter stenosis (%)	38.5 (35.2–42.8)	39.7 (35.6–43.5)	37.4 (34.9–39.1)	36.9 (34.3–39.3)	0.89^†^ 0.47^††^
Late luminal loss^b^ (mm)		0.03 (− 0.07 to 0.20)		0.05 (− 0.07 to 0.23)	0.89^††c^
Minimal lumen area (mm^2^)	5.38 (4.48–6.71)	5.84 (4.90–6.66)	5.77 (5.04–6.49)	5.57 (4.90–6.44)	0.88^†^ 0.47^††^
Neointimal volume (mm^3^)	16.3 (10.2–22.9)	16.8 (10.9–24.5)	10.5 (6.8–16.0)	9.7 (6.8–12.6)	0.91^†^ 0.25^††^
Stent segment vessel volume (mm^3^)	431.5 (248.9–613.8)	463.0 (260.0–617.8)	253.9 (198.8–516.6)	310.6 (206.7–487.8)	0.11^†^ 0.57^†^
Stent segment lumen volume (mm^3^)	204.5 (101.9–249.1)	202.2 (100.2–256.3)	119.2 (80.9–231.4)	138.5 (82.9–201.7)	0.87^†^ 0.95^††^
Stent segment plaque volume (mm^3^)	227.0 (148.2–359.0)	249.5 (159.8–392.2)	145.0 (106.9–285.3)	185.3 (109.1–286.0)	0.02^†^ 0.42^†^

Data are given as medians with 95% confidence intervals in parenthesis. No significant differences within or between groups

^†^Changes by time in both groups from baseline to follow-up

^††^Differences between the groups from baseline to follow-up (interaction effect)

^a^ANOVA (analysis of variance) for repeated measures was used to analyze baseline and follow-up data

^b^Late luminal loss (the angiographic minimal lumen diameter after PCI minus the minimal lumen diameter at follow-up) was only measured at follow-up

^c^Mann–Whitney *U* test

**Table 4:** Cardiorespiratory variables at baseline and follow-up

	AIT		MCT		*P* value^a^
	Baseline	Follow-up	Baseline	Follow-up	
Resting heart rate (beats/min)	62 (56–65)	59 (54–64)	58 (53–65)	59 (54–64)	0.30^†^ 0.36^††^
Peak heart rate (beats/min)	157 (147–166)	161 (153–168)	153 (143–163)	155 (144–165)	0.19^†^ 0.37^††^
Heart rate recovery, 1 min^b^	28 (23–33)	30 (26–36)	27 (24–31)	30 (26–34)	0.04^†^ 0.76^††^
Peak oxygen uptake (mL*kg^−1^*min^−1^)	30.6 (28.9–32.6)	34.2 (32.1–36.2)	29.1 (25.6–33.6)	31.1 (27.6–35.5)	< 0.001^†^ 0.15^††^

Data are given as medians with 95% confidence intervals in parenthesis

^†^Changes by time in both groups from baseline to follow-up

^††^Difference between the groups from baseline to follow-up (interaction effect)

^a^ANOVA analysis of variance for repeated measures was used to analyze baseline and follow-up data

^b^Subtracted 1 min heart rate from the heart rate immediately after ended exercise

## Discussion

We assessed the EVR and in-stent geometry in patients undergoing two different aerobic exercise interventions following implantation of second-generation DES. Our main findings were the demonstration of decreased plaque burden and necrotic core in the distal stent edges in both groups, and with a larger decrease in the AIT group. No harmful effects, i.e. plaque growth, increased necrotic core content, or increased in-stent neointimal volume, were demonstrated after exercise intervention.

We are not aware of previous studies assessing exercise-induced effects in-stent edges after DES implantation. Our results are in contrast to studies of the natural history of the EVR following implantation of second-generation DES [18–20]. These differences may be related to exercise effects. The 2-year follow-up SPIRIT II substudy found a significant plaque area decrease in proximal stent edges, representing the only trial that has demonstrated significant plaque changes in-stent edges at follow-up [20]. The SPIRIT III JAPAN study found evidence of expansive remodeling in the distal stent edges [19], but no changes in plaque volumes, and the SPIRIT III US study demonstrated a significant lumen volume reduction in the proximal stent edges with no evidence of plaque changes [19]. Finally, the ENDEAVOR II and III studies did not find any significant plaque changes in the proximal or distal stent edges [18]. In contrast to the abovementioned natural history studies, we demonstrated a decreased plaque burden and necrotic core content in the distal stent edges, with no significant changes in the proximal stent edges.

With respect to in-stent geometry, three previous studies have assessed exercise-induced effects on coronary stents using coronary angiography, demonstrating reduced late luminal loss in the exercise groups at follow-up [9–11]. Munk et al. found that 6 months of AIT reduced late lumen loss both in patients treated with DES and bare-metal stents compared to inactive patients [9]. Similar results were obtained in two different studies including patients receiving both first- and second-generation DES. After 9 months in a cardiac rehabilitation exercise program, a reduced late luminal loss rate was demonstrated in the exercise group [10, 11]. We did not demonstrate any significant late luminal loss, stent segment vessel volume reduction or neointimal volume increase in the stented segment between or within exercise groups. However, our study did not include an inactive control group for comparison and, therefore, we cannot fully exclude an effect from exercise on in-stent geometry. One may also speculate that a prolonged exercise intervention could have induced significant in-stent changes or altered findings with respect to plaque changes in-stent edges. We observed a significant increase in-stent segment plaque volume in both groups, with no changes between the groups. The increase did not affect luminal parameters, and is, therefore, of uncertain importance.

During aerobic exercise, coronary WSS increases as the cardiac output and coronary blood flow increase [21, 22]. It is hypothesized that increased exercise-induced WSS in coronary arteries may induce an anti-atherogenic phenotype in the coronary endothelium [23] via altered gene expression and increased endothelium-derived nitrogen oxide synthase phosphorylation [24]. In contrast to this hypothesis, a fluid dynamic model demonstrated a significant increase in necrotic core content in coronary segments with high WSS, implicating a phenotype transformation into more vulnerable plaques [25].

Even though exercise generally increases WSS in the coronary circulation, individual vessel geometry may result in unique WSS patterns for each patient. Thus, local factors may also contribute to the demonstrated differences in the EVR in natural history studies and this study. Theoretically, low WSS with consequently more plaque formation prior to stenting in the distal lesion area could allow for a greater potential for exercise-induced changes in the distal compared to the proximal stent edge [26]. We hypothesized that WSS during exercise would increase more in the AIT group compared to the MCT due to differences in exercise intensity. Although our findings must be interpreted with caution, this may explain the larger reductions in plaque burden and necrotic core content in distal stent edges in the AIT group compared to MCT.

Based on our findings in the current study and the previous published paper from this trial [12], it seems probable that aerobic exercise has a potential to induced beneficial changes in coronary atherosclerosis. Thus, our findings strengthen the scientific evidence of recommending aerobic exercise in CAD patients treated with PCI and stent implantation, and argue for increased use of exercise as medicine. One may also speculate if AIT is superior to MCT with respect to beneficial intracoronary changes after exercise. However, our results from this small trial must be interpreted with caution, and larger trials are warranted to confirm our results.

### Limitations

Our study has some obvious limitations. The small sample size is an important limitation due to low statistical power. However, being the first randomized exercise trial assessing EVR and in-stent geometry, we believe these data are of value. The use of an inactive control group was discussed prior to study inclusion, but was considered unethical due to the well-documented benefits of exercise in CAD patients. Furthermore, all patients undergoing PCI at our hospital are offered cardiac rehabilitation as a part of the routine. In our trial, device-related inconsistencies are minimized when analyzing the same generation of DES, although differences in drug release kinetics, drug concentration and strut thickness varying within the same generation of DES might have an impact on the EVR results [27, 28]. Furthermore, methodological inconsistencies when obtaining the IVUS data could alter the precise EVR assessment, even though previous studies have shown high reproducibility of IVUS data in both stented and non-stented segments [29–32]. RF-IVUS also has some general limitations with respect to tissue characterization, including poor axial resolution and the use of post-processing algorithms instead of direct measurement of plaque components. Finally, relatively short period of exercise intervention is a limitation with respect to long-term effect on EVR after DES implantation.

## Conclusion

This small study of patients undergoing percutaneous coronary intervention with DES implantation demonstrated decreased plaque burden and necrotic core content in the distal stent edges, with larger reductions in the high-intensity exercise group. No harmful effects, i.e. plaque growth, increased necrotic core content, or increased in-stent neointimal volume, were demonstrated. Our results need to be confirmed in a larger population.
